# Cerebral Substrates for Controlling Rhythmic Movements

**DOI:** 10.3390/brainsci10080514

**Published:** 2020-08-03

**Authors:** Naho Konoike, Katsuki Nakamura

**Affiliations:** Section of Neuroscience, Primate Research Institute, Kyoto University, Kyoto 484-8501, Japan; nakamura.katsuki.4z@kyoto-u.ac.jp

**Keywords:** rhythm, finger-tapping, brain imaging, patient study, neuroscience

## Abstract

Our daily lives are filled with rhythmic movements, such as walking, sports, and dancing, but the mechanisms by which the brain controls rhythmic movements are poorly understood. In this review, we examine the literature on neuropsychological studies of patients with focal brain lesions, and functional brain imaging studies primarily using finger-tapping tasks. These studies suggest a close connection between sensory and motor processing of rhythm, with no apparent distinction between the two functions. Thus, we conducted two functional brain imaging studies to survey the rhythm representations relatively independent of sensory and motor functions. First, we determined brain activations related to rhythm processing in a sensory modality-independent manner. Second, we examined body part-independent brain activation related to rhythm reproduction. Based on previous literature, we discuss how brain areas contribute rhythmic motor control. Furthermore, we also discuss the mechanisms by which the brain controls rhythmic movements.

## 1. Introduction

As Fraisse (1984) highlighted, rhythm is difficult to define, but in general, rhythm refers to the ordered chronological patterns perceived between events (e.g., sounds and sounds) [1]. The basic components of rhythm include “pulses” that consist of the same stimulus at equal intervals, “meters” that are formed by accents on the strength and weakness of sounds, and “beats” which are time units of a certain period that are perceived by the listener due to the presence of a meter. A continuous stimulus is perceived as grouping by a metrical structure or repetition, that is, “rhythm” [2]. Thus, different combinations of various factors, such as the interval between stimuli, intensity patterns, and the presence or absence of repetition, will result in differently perceived rhythms. Although “rhythm” is a fundamental element of music, it is also important in body movements including speaking (“verbal rhythm” and “rhythmic movement”). Many people experience their bodies move naturally when they listen to music, and rhythm perception and movement are closely related. It is not difficult to imagine that the movements of dance and speech are rhythmic, but even in movements without music, such as sports, a series of smooth movements can only be made if each movement is performed at the appropriate time.

For decades, many researchers have attempted to elucidate the neural mechanisms that underlie rhythmic motor control. Most of these studies are psychophysical studies that use two kinds of finger-tapping task for humans; one is the “rhythm synchronization task” in which the participants are required to tap their finger synchronously with external/sensory cues, and the another is the “rhythm reproduction task”, in which the participants memorize a specific rhythmic pattern and reproduce it by tapping. These tasks have been used to elucidate the characteristics of rhythm information processing in humans. In addition to these psychophysiological studies, there is a long history of neuropsychological studies on patients with Parkinson’s disease (PD) or brain injury. These studies have suggested that the frontal cortex (including the premotor cortex, supplementary motor area (SMA)), basal ganglia, and cerebellum are involved in controlling rhythmic movements. In recent decades, functional brain imaging studies for rhythmic motor control in humans have been conducted intensively [3,4,5,6,7,8,9,10,11,12,13,14,15]. These brain imaging studies have revealed the neural substrates involved in rhythmic motor control using tapping tasks.

Here, we summarize and review neuropsychological studies on rhythmic movement deficits, as well as brain imaging studies related to the neural basis of rhythmic motor control and rhythm information processing. We subsequently discuss how each brain region contributes to the control of rhythmic movements.

## 2. Brain Regions Responsible for Rhythmic Movements

### 2.1. Patient Studies

Early neuropsychological studies have suggested that the frontal cortex, basal ganglia, and cerebellum are involved in rhythmic tapping. Halsband et al. (1993) reported behavioral features of rhythm synchronization and reproduction tasks using the right index finger in patients with unilateral SMA lesions due to cerebral infarction [16]. The authors found that patients with left hemisphere lesions involving the SMA showed an increase in error in the reproduction task, while, interestingly, no performance deficits were observed in the synchronization task, showing that the movement itself was not impaired. This study suggests that the crucial role of SMA is in rhythm reproduction, and not in rhythm synchronization. Thus, the SMA may be involved in motor control based on internal rhythm, and not in controlling movement itself.

Picton et al. (2006) investigated the behavioral performance of patients with focal lesions in the frontal cortex in terms of rhythm synchronization and reproduction tasks at constant intervals [17]. The patients were divided into four groups based on the location of the lesion in the frontal lobe: left lateral, right lateral, inferior medial, and superior medial groups. The authors found that the patients with lesions to the right lateral frontal cortex, particularly to the ventral premotor cortex, showed an abnormally high variability in the timing of tapping with the right index finger for both the rhythm synchronization and reproduction tasks. Halsband et al. (1993) also reported that patients with lesions in the premotor cortex showed impairment of both the rhythm reproduction and synchronization tasks [16]. These two studies suggest that the premotor cortex is involved in controlling rhythmic movement itself.

Patients with PD show a variety of movement disorders, including tremor, posture, and gait impairments. Regarding rhythm generation, in the 1980s, a clinical report described a patient with PD who was impaired in performing finger-tapping tasks [18]. Since then, disability of rhythm production in patients with PD has been studied in earnest, and impairments in the performance of repetitive motor tasks, such as finger-tapping and wrist rotation, have been reported [19,20,21,22,23,24,25,26]. For example, Pastor et al. (1992) used a task in which participants were required to perform flexion-extension wrist rotation at constant rates of 0.5, 1, 1.5, 2, and 2.5 Hz [24]. The authors found that patients with PD showed both less accuracy and greater variability in response intervals when performing repetitive wrist rotation at 2 and 2.5 Hz compared to healthy controls; these results suggest that the basal ganglia are involved in controlling rhythmic movements on a millisecond scale. Moreover, they also reported improvement in these impairments after dopamine administration.

Studies of focal lesions in the basal ganglia also provide information about the function of this area in controlling rhythmic movements. Schwartze et al. (2011) reported the performance on rhythm perception and reproduction in patients with damage to the basal ganglia due to stroke [27]. The participants were asked to perform a synchronization tapping task, where they had to align finger taps to tone sequences containing a tempo acceleration or tempo deceleration, and to continue tapping at the final tempo without stimuli. The authors found that damage to the basal ganglia induced more heterogeneous distribution of individual rates, as well as more variable tapping during this adaptive synchronization-reproduction task. These results confirm that lesions of the basal ganglia affect the ability to perceive and reproduce rhythm. The basal ganglia reportedly function in detecting beat or metric structure of rhythm and controlling its reproduction.

While the link between PD and the deficits of finger-tapping movements has suggested the function of the basal ganglia in rhythmic movement, some studies have provided contradictory results. For example, Spencer and Ivry (2005) reported PD patients with no impairment in the performance of finger-tapping movements [28], while Aparicio et al. (2005) reported no significant difference in performance of a finger-tapping task between the healthy control group and patients with brain damage in the left basal ganglia [29]. PD is a progressive neurodegenerative disease, and the patients are in different disease stages and under different medications. Thus, inconsistency of the results would be, to some extent, due to the variability of the patient’s condition. Several functional imaging studies have elucidated the role of the basal ganglia for controlling beat-based timing [30,31,32]. Overall, we believe that the basal ganglia play an important role in controlling rhythmic movements.

The contribution of the cerebellum in timing control has been studied in the context of brain damage [22,28,33,34,35,36,37]. Spencer et al. (2003) focused on controlling discrete versus continuous movements [37]; patients with unilateral cerebellar lesions due to tumor or stroke were required to perform two motor tasks, circle drawing and finger-tapping. For a “continuous” circle drawing, the participants were instructed to draw circles continuously and smoothly, whereas for an “intermittent (or discrete)” circle drawing, participants were required to pause after drawing each circle. The continuous and discrete manners were the same as those in the finger-tapping task. The results showed that the temporal variability of performance by the contralateral limb was not significantly different from that of the ipsilateral limb when producing continuous movements, regardless of the type of movement (circle drawing or finger-tapping). In contrast, the cycle duration in discrete movements was more variable in the contralateral limb than in the ipsilateral limb. This study suggested that the cerebellum is essential for controlling discrete movements that require explicit timing control but is not essential for continuous movements. Afterward, Grube et al. (2020) have revealed the cerebellum is involved in the duration-based timing processing. The authors used two auditory timing tasks to require the patients with cerebellar degeneration to discriminate absolute time of single intervals and to discriminate beat-based patterns. The patients showed greater impairments of the performance in the absolute timing task compared to controls [38]. The results are consistent with several reports in brain imaging study in healthy participants [30] and a trans magnetic stimulation study [39]. These results suggest the cerebellar contribution to the absolute timing of single sub-second intervals.

While patient studies can directly assess the function of a brain region on rhythmic motor control, they have several limitations, including the inability to control the extent of damage in the target region, and the difficulty of assessing function in terms of rhythm alone due to complications of other impairments. Thus, it is necessary to have a combined perspective using different research methods, such as patient studies and brain functional imaging studies.

### 2.2. Brain Imaging Studies

More than 20 years ago, Rao et al. (1997) revealed brain regions involved in controlling motor timing using functional magnetic resonance imaging (fMRI) in healthy volunteers [10]. In this experiment, the participants performed four tasks: (1) listening, (2) synchronization, (3) reproduction, and (4) discrimination of constant auditory stimuli with 300-ms or 600-ms intervals. As a result, activation in the medial frontal lobe, including the SMA, basal ganglia, and cerebellum was only observed during the reproduction task, while activation in the SMA was also observed in the interval discrimination task; similar findings were reported by Bengtsson et al. (2004) [40]. The authors used two tasks in which the participants were required to (1) reproduce learned rhythm sequences by tapping with one finger (rhythm reproduction task), and (2) tap with five fingers in learned orders of the fingers at an equal interval (order reproduction task). Brain activity during tapping was measured by fMRI. The authors found that—in addition to the SMA, basal ganglia, and cerebellum—the premotor cortex and the bilateral superior temporal gyrus (STG) were more activated in the rhythm reproduction task than in the order reproduction task. These two studies show common results in that the SMA-basal ganglia-cerebellum network is involved in controlling tapping movements. Activation in the basal ganglia is observed related to rhythm processing, especially for beat perception of rhythm [30,31,32], thus the network may also be involved in controlling beat-based timing. In addition to the network, activation in the premotor cortex and STG were observed in the study by Bengtsson et al. (2004) that utilized more complex rhythmic sequences than those used by Rao et al. (1997). Thus, the premotor cortex and STG may function in information processing of complex time-series patterns.

Earlier studies have discussed the function of the cerebellum as a timekeeper system [22]. Indeed, patients with cerebellar lesions showed deficits in the discrimination of time duration [22] or increased variability during repetitive finger-tapping [37]. Cerebellar activations were also reported in brain imaging studies in which participants were requested to discriminate the absolute time duration [30,41,42,43]. Furthermore, several studies have suggested a role for the cerebellum in auditory rhythm information processing [44,45,46,47,48,49]. In addition to these reports, we found cerebellum activation during the participants perceived and memorized visual sequence, as well as during the auditory sequence [7]. Our results are consistent with the involvement in sensory input of rhythm, regardless of sensory modalities such as visual, auditory, and tactile [14,46]. Together all, these results suggest that the cerebellum plays a crucial role in the storage of absolute durations in rhythm sequence in a multimodal manner.

## 3. Rhythm Representation in the Brain

So far, we have reviewed the previous literature that revealed the brain regions underlying rhythmic motor control. However, to reproduce rhythmic movements, we should perceive the temporal organization of a series of events (often auditory stimuli), represent it as rhythm, transform the rhythm to the temporal sequence of series of movements, and output it as rhythmic movements. Thus, it is difficult to distinguish brain areas related to the control of rhythmic movement from the sensory and motor information itself. We hypothesized that rhythm information can be represented by the temporal sequence of stimuli, which should be independent of sensory modality. However, the rhythm information is also represented by the temporal sequence of movements, which should be independent of body parts (muscles per se), such as the finger or foot. Thus, we challenged the dissociation and elucidation of rhythm representation in our two fMRI studies.

**Figure 1:**
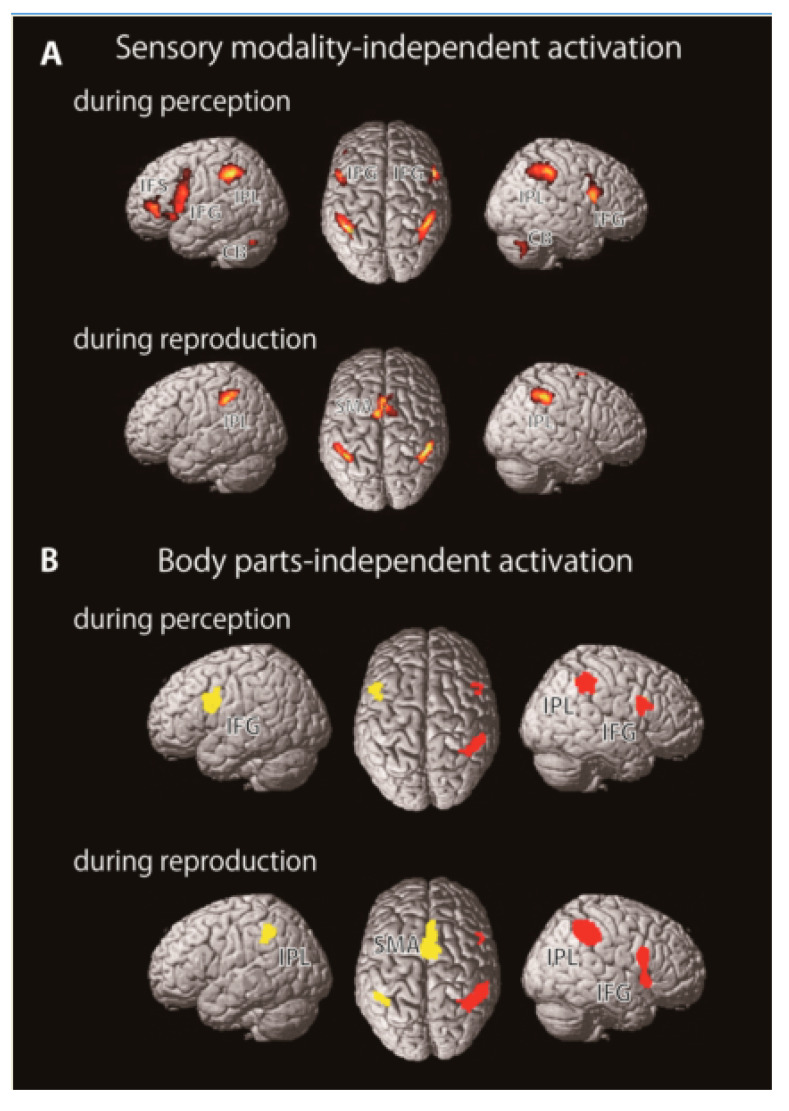
(**A**) Sensory modality-independent rhythm representation. Brain regions commonly activated during auditory and visual rhythm tasks. (**B**) Body part-independent rhythm representation. Red and yellow areas indicate the cortical regions which were activated by all three motor effectors. Abbreviations: CB: Cerebellum, IFG: Inferior frontal gyrus, IFS: Inferior frontal sulcus, IPL: Inferior parietal lobule, SMA: Supplementary motor area (A: modified from Konoike et al., 2012, B: modified from Konoike et al., 2015).

First, we attempted to elucidate neural substrates representing rhythm independent of sensory modalities. Almost all brain imaging studies had used auditory stimuli in rhythm synchronization and reproduction tasks. We believe that it is important to note neural activations regardless of sensory modality of stimuli. The brain substrates exhibiting the activations must be responsible for the representation of temporal organization of sensory stimuli as rhythm, that is, rhythm representation. We measured brain activity by fMRI while healthy participants perceived, memorized, and reproduced visual, as well as auditory, rhythmic sequences by finger-tapping. Regardless of the sensory modalities (visual or auditory) of the rhythmic stimulus, we found activation in the inferior frontal gyrus (IFG), inferior parietal lobule (IPL), SMA, and cerebellum (Figure 1, [7]). These areas are thought to be involved in representing the temporal sequence of rhythm, and not in sensory processing itself. We have also revealed a crucial role of functional connectivity between the auditory cortex and the premotor cortex when we used the auditory rhythmic sequences [6]. The results suggest that the importance of auditory-motor interaction in rhythmic processing, especially for perception and reproduction of auditory rhythm and it is consistent with previous brain imaging studies [31,50].

Several brain imaging studies have focused on brain activity while simply listening to rhythm, and found that listening to rhythms activates motor areas of the brain. For example, Chen et al. (2008) used a rhythm listening task to examine the brain regions related to rhythm perception [51]. In their perceptual task, the participants simply passively listened to the rhythm and were not required to make any motor response. The authors reported activation in motor brain regions, such as the SMA, premotor cortex, and cerebellum. The authors have reported the activation changes in the premotor cortex and cerebellum related to listening to auditory rhythms with anticipation. The premotor cortex and cerebellum were also recruited in synchronization tapping to the same rhythms, these suggest the overlapping of a neural system between expectancy for rhythm perception and rhythm production. Bengtsson et al. (2009) also reported that the pre-SMA, premotor cortex, and cerebellum were active while listening to rhythms without any motor response [52]. The authors prepared three types of rhythmic stimuli: isochronous pattern (sound with constant rate), complex rhythms (with metric structure), and more complex rhythms (without metric structure), in addition to a random sequence. When listening to the rhythm pattern, significant activations in the pre-SMA, dorsal premotor cortex, and cerebellum were observed compared to listening to the random sequence. In addition, the activity of the pre-SMA significantly increased in the isochronous and metric rhythms, which are simpler and externally triggered patterns; these activations may reflect more precise predictions of timing.

**Figure 2:**
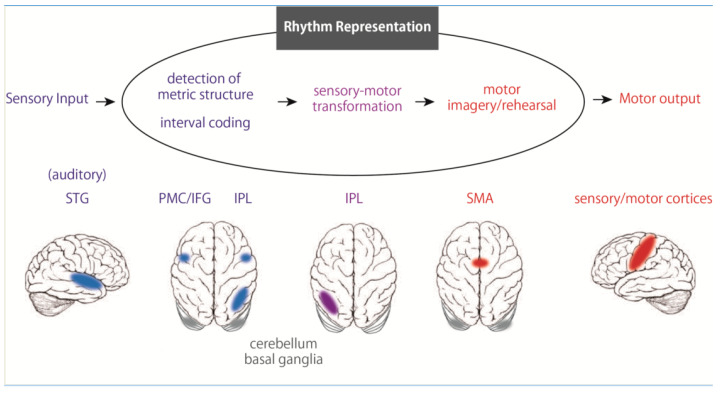
Hypothetical model of rhythm processing after sensory input, the premotor cortex (PMC)/inferior frontal gyrus (IFG) and inferior parietal lobule (IPL) receive auditory rhythm information from the superior temporal gyrus (STG). The sensory rhythm information is stored in the fronto-parietal network. During rhythm reproduction, the IPL transforms the temporal information into the motor sequence of the rhythm, and the supplemental motor area (SMA) reproduces the rhythm through various body parts while monitoring the output with the feedback signals from the somatosensory/motor cortices. Subcortical regions including the basal ganglia and cerebellum also play an important role in rhythm processing.

Next, to survey the neural substrates for rhythm representation, we measured brain activity by fMRI while participants memorized rhythms and reproduced them by tapping with the right finger, left finger, or foot. We found significant activations, regardless of the body parts, during rhythm perception and reproduction in the IFG, IPL, and SMA (Figure 2, [6]). Although the activation peak of the IFG observed here was restricted to the opercular region (BA 44), the activation extended and was involved in the premotor cortex. The IFG is implicated in syntax processing of language and music [53,54,55], as well as in memory of structured sequences [56]. Together with previous findings, the IFG plays a crucial role in organizing perceived sound elements into a structured temporal sequence of rhythm. In contrast, some brain imaging studies have suggested the involvement of the IPL regions in time perception [57,58], especially that of the right IPL in temporal prediction and production [59]. In addition, dysfunction of the IPL can induce impairment in temporal information processing [20,60,61,62]. Furthermore, Grahn and Rowe (2013) reported activation in the IPL during the detection of a musical beat in irregular rhythms that require memorization of each interval, unlike that of regular rhythms [63]. These results suggest that the IPL is involved in representing the temporal information of rhythm, especially in the perceptual time of each interval of rhythm sequences. Importantly, these areas showed rhythm-related activations independent of the body parts, but also independent of sensory modalities, including auditory or visual [7]. Thus, the IFG, IPL, and SMA may play a central role in rhythm representation (Figure 2). A recent review of acquired amusia after stroke provides important information about neural substrates underlying rhythm processing [64]. As a result of the compilation of numerous previous case studies, not only the STG but also the IFG and IPL have been shown to be core regions for acquired amusia, which is consistent with our model of rhythmic motor control.

## 4. Temporal Information Processing

There are many cognitive models of temporal information processing. Ivry and Schlerf have classified these models into two models: the dedicated model and the intrinsic model [65,66,67].

The dedicated model is a model that assumes a specialized mechanism for processing temporal information, an internal clock. The dedicate model includes the scalar timing model [68], cerebellar timing model [22] and striatal-beat-frequency model [67]. During a time (duration) discrimination, this model consists of three processes: a clock process, a memory process, and a decision process. In the clock process, the pacemaker sends pulses at regular intervals to the accumulator. In the accumulator, pulse trains are converted to number of pulses (time duration). Then, the information is stored working memory, which is temporal memory. The number of pulses is stored in reference memory through training. In the decision process, when we perceive stimulation, the pacemaker sends pulses during the stimulus, to the accumulator. The number of pulses stored in current working memory is compared with the time information in reference memory, and it is judged. The dedicated model is a conceptual hypothesis. Based on the results of neurophysiological experiments, an expanded model is proposed. Neural oscillation of cortical or subcortical neurons functions as a pacemaker. For example, in the striatal-beat-frequency model, timing is based on the coincidental activation of spiny neurons in the basal ganglia by cortical neural oscillations [67,69].

In contrast, the intrinsic model, which has attracted much attention in recent years, hypothesizes that temporal information is encoded by dynamic changes in neurons, rather than by mechanisms specific to temporal information. For example, the idea is that the duration of a visual stimulus is perceived depending on dynamic changes in neurons in the visual cortex, and the duration of an auditory stimulus is encoded by changes in neurons in the auditory cortex. In this intrinsic model, temporal information is thought to change in response to circumstances such as the modality and time scale of the stimulus, which is a property called “context dependence”. This intrinsic model hypothesis is supported by computer simulation experiments that show that local networks of neurons can convey temporal information through context-dependent changes over time [70].

Recently, a hybrid model has been proposed [71,72], which consists of main core timing system and context-specific system. We have reported the rhythm-related brain areas regardless of sensory modality [7] or body parts [6], suggests the existence of the main core timing system. Further discussion is expected on the mechanism by which rhythmic information is processed in the brain.

## 5. Outlook

Time is a sense that, unlike audition and vision, has no specific receptors. Hence, the mechanism involved in processing the time information by the brain is poorly understood. When considering the mechanisms of time perception and timing control, we must consider the “timescale”. Time can be broadly classified into “circadian timing,” which is a 24-h cycle, “interval timing,” which ranges from a few milliseconds to a few seconds, and “millisecond timing,” which is a few milliseconds [66,67]. Lewis and Miall (2003) argued that different brain regions and different control mechanisms are involved in different timescales [8]. The authors surveyed a number of previous functional imaging studies and reported that time perception and motor tasks in the millisecond range were associated with activation in the motor control regions, such as the cerebellum, basal ganglia, SMA, and premotor cortex, while in second-time ranges, activation in the prefrontal cortex and parietal cortex were found to be related to behavioral tasks. Thus, they proposed that an automatic timing mechanism operates for a short time range (milliseconds), whereas, for a time range of seconds, a conscious control mechanism exists due to attentional and cognitive intervention. Previously, we trained two Japanese macaques to press a button repetitively in response to external cues and have reported the behavioral features [73]. When the cue-intervals were constant and sub-second, the monkey’s button press was predictive. However, when the cue-intervals were on a supra-second scale, the reaction time of the monkeys did not differ from those to the cues at random intervals. These results suggest that when the movements are constant and with shorter intervals, the automatic timing system may be recruited, whereas in the movements with longer intervals, unlike humans, the conscious control mechanism does not function. It is consistent with the idea of the hybrid model that distinct mechanisms work at different time scale [71,72].

As described above, research on the brain mechanisms of rhythm perception and production has been mainly conducted in humans. Humans naturally move their bodies upon hearing music; this sensory-motor interaction is thought to be unique to humans, but some studies have shown that animals such as parrots, rhesus macaques, Japanese macaques, chimpanzees and sea lions can exhibit rhythmic body movements synchronized to music [73,74,75,76,77]. Patel et al. (2009) analyzed the movements of the famous parrot named Snowball, who dances to pop music, and reported that its rhythmic movements synchronized to external musical stimuli [75]. These reports suggest that synchronized movements to external stimuli do not represent a uniquely human ability, and that other animals are also capable of these movements. As long as the experiment is performed on human participants, the experiment is limited to noninvasive methods such as psychophysical, neurophysiological or brain imaging. Therefore, it is not possible to directly answer the question of the role that each brain region plays in rhythm information processing. To elucidate the detailed neural mechanisms of rhythm perception and production, electrophysiological studies using monkeys with brains close to humans will likely provide further information. In this sense, the series of neurophysiological studies by Merchant and his colleagues could lead to a greater understanding of the neuronal network for rhythmic motor control at higher temporal and spatial resolutions [78,79,80].

Furthermore, some attempts have been made to utilize the close relationship between auditory rhythm and movement to apply it to therapy. Thaut (2005) systematized neurologic music therapy, in which music is used as a therapeutic intervention in medical care, such as in sensorimotor and cognitive rehabilitation [81]. Although it has not yet taken root in the Japanese medical field, this kind of rehabilitation based on neuroscientific findings is likely to attract attention in the future [82].

## References

[1] Fraisse P. (1984). Perception and estimation of time. Annu. Rev. Psychol..

[2] Cooper G.W., Meyer L.B. (1960). The Rhythmic Structure of Music.

[3] Bednark J.G., Campbell M.E., Cunnington R. (2015). Basal ganglia and cortical networks for sequential ordering and rhythm of complex movements. Front. Hum. Neurosci..

[4] Bengtsson S.L., Ehrsson H.H., Forssberg H., Ullén F. (2005). Effector-independent voluntary timing: Behavioural and neuroimaging evidence. Eur. J. Neurosci..

[5] De Manzano Ö., Ullén F. (2012). Activation and connectivity patterns of the presupplementary and dorsal premotor areas during free improvisation of melodies and rhythms. Neuroimage.

[6] Konoike N., Kotozaki Y., Jeong H., Miyazaki A., Sakaki K., Shinada T., Sugiura M., Kawashima R., Nakamura K. (2015). Temporal and Motor Representation of Rhythm in Fronto-Parietal Cortical Areas: An fMRI Study. PLoS ONE.

[7] Konoike N., Kotozaki Y., Miyachi S., Miyauchi C.M., Yomogida Y., Akimoto Y., Kuraoka K., Sugiura M., Kawashima R., Nakamura K. (2012). Rhythm information represented in the fronto-parieto-cerebellar motor system. Neuroimage.

[8] Lewis P.A., Miall R.C. (2003). Distinct systems for automatic and cognitively controlled time measurement: Evidence from neuroimaging. Curr. Opin. Neurobiol..

[9] Ramnani N., Passingham R.E. (2001). Changes in the human brain during rhythm learning. J. Cogn. Neurosci..

[10] Rao S.M., Harrington D.L., Haaland K.Y., Bobholz J.A., Cox R.W., Binder J.R. (1997). Distributed neural systems underlying the timing of movements. J. Neurosci..

[11] Schwartze M., Rothermich K., Kotz S.A. (2012). Functional dissociation of pre-SMA and SMA-proper in temporal processing. Neuroimage.

[12] Xu D., Liu T., Ashe J., Bushara K.O. (2006). Role of the olivo-cerebellar system in timing. J. Neurosci..

[13] Jerde T.A., Childs S.K., Handy S.T., Nagode J.C., Pardo J.V. (2011). Dissociable systems of working memory for rhythm and melody. Neuroimage.

[14] Karabanov A., Blom O., Forsman L., Ullén F. (2009). The dorsal auditory pathway is involved in performance of both visual and auditory rhythms. Neuroimage.

[15] Witt S.T., Laird A.R., Meyerand M.E. (2008). Functional neuroimaging correlates of finger-tapping task variations: An ALE meta-analysis. Neuroimage.

[16] Halsband U., Ito N., Tanji J., Freund H.-J. (1993). The role of premotor cortex and the supplementary motor area in the temporal control of movement in man. Brain.

[17] Picton T.W., Stuss N.T., Shallice T., Alexander M.P., Gillingham S. (2006). Keeping time: Effects of focal frontal lesions. Neuropsychologia.

[18] Frischer M. (1989). Voluntary vs. autonomous control of repetitive finger tapping in a patient with Parkinson’s disease. Neuropsychologia.

[19] Freeman J.S., Cody F.W., Schady W. (1993). The influence of external timing cues upon the rhythm of voluntary movements in Parkinson’s disease. J. Neurol. Neurosurg. Psychiatry.

[20] Harrington D.L., Haaland K.Y., Knight R.T. (1998). Cortical Networks Underlying Mechanisms of Time Perception. J. Neurosci..

[21] Ivry R.B. (1986). Force and timing components of the motor program. J. Mot. Behav..

[22] Ivry R.B., Keele S.W. (1989). Timing functions of the cerebellum. J. Cogn. Neurosci..

[23] O’Boyle D.J., Freeman J.S., Cody F.W. (1996). The accuracy and precision of timing of self-paced, repetitive movements in subjects with Parkinson’s disease. Brain.

[24] Pastor M.A., Jahanshahi M., Artieda J., Obeso J.A. (1992). Performance of repetitive wrist movements in parkinson’s disease. Brain.

[25] Wing A.M., Keele S., Margolin D.I. (1984). Motor Disorder and the Timing of Repetitive Movements. Ann. N. Y. Acad. Sci..

[26] Wing A.M., Miller E. (1984). Basal ganglia lesions and psychological analyses of the control of voluntary movement. Ciba Found. Symp..

[27] Schwartze M., Keller P.E., Patel A.D., Kotz S.A. (2011). The impact of basal ganglia lesions on sensorimotor synchronization, spontaneous motor tempo, and the detection of tempo changes. Behav. Brain Res..

[28] Spencer R.M., Ivry R.B. (2005). Comparison of patients with Parkinson’s disease or cerebellar lesions in the production of periodic movements involving event-based or emergent timing. Brain Cogn..

[29] Aparicio P., Diedrichsen J., Ivry R.B. (2005). Effects of focal basal ganglia lesions on timing and force control. Brain Cogn..

[30] Teki S., Grube M., Kumar S., Griffiths T. (2011). Distinct neural substrates of duration-based and beat-based auditory timing. J. Neurosci..

[31] Grahn J.A., Rowe J.B. (2009). Feeling the beat: Premotor and striatal interactions in musicians and nonmusicians during beat perception. J. Neurosci..

[32] Grahn J.A. (2009). The role of the basal ganglia in beat perception: Neuroimaging and neuropsychological investigations. Ann. N. Y. Acad. Sci..

[33] Bo J., Block H.J., Clark J.E., Bastian A.J. (2008). A cerebellar deficit in sensorimotor prediction explains movement timing variability. J. Neurophysiol..

[34] Ivry R.B., Keele S.W., Diener H.C. (1988). Dissociation of the lateral and medial cerebellum in movement timing and movement execution. Exp. Brain Res..

[35] Schlerf J.E., Spencer R.M.C., Zelaznik H.N., Ivry R.B. (2007). Timing of rhythmic movements in patients with cerebellar degeneration. Cerebellum.

[36] Schwartze M., Keller P.E., Kotz S.A. (2016). Spontaneous, synchronized, and corrective timing behavior in cerebellar lesion patients. Behav. Brain Res..

[37] Spencer R.M., Bjedov I., Tenaillon O., Gérard B., Souza V., Denamur E., Radman M., Taddei F., Matic I. (2003). Disrupted timing of discontinuous but not continuous movements by cerebellar lesions. Science.

[38] Grube M., Cooper F.E., Chinnery P.F., Griffiths T.D. (2010). Dissociation of duration-based and beat-based auditory timing in cerebellar degeneration. Proc. Natl. Acad. Sci. USA.

[39] Grube M., Lee K.-H., Griffiths T.D., Barker A.T., Woodruff P.W. (2010). Transcranial Magnetic Theta-Burst Stimulation of the Human Cerebellum Distinguishes Absolute, Duration-Based from Relative, Beat-Based Perception of Subsecond Time Intervals. Front. Psychol..

[40] Bengtsson S.L., Ehrsson H.H., Forssberg H., Ullén F. (2004). Dissociating brain regions controlling the temporal and ordinal structure of learned movement sequences. Eur. J. Neurosci..

[41] Belin P., McAdams S., Thivard L., Smith B., Savel S., Zilbovicius M., Samson S., Samson Y. (2002). The neuroanatomical substrate of sound duration discrimination. Neuropsychologia.

[42] Rao S.M., Mayer A.R., Harrington D.L. (2001). The evolution of brain activation during temporal processing. Nat. Neurosci..

[43] Mathiak K., Hertrich I., Grodd W., Ackermann H. (2004). Discrimination of temporal information at the cerebellum: Functional magnetic resonance imaging of nonverbal auditory memory. Neuroimage.

[44] Griffiths T.D., Johnsrude I., Dean J.L., Green G.G.R. (1999). A common neural substrate for the analysis of pitch and duration pattern in segmented sound?. Neuroreport.

[45] Ivry R.B., Spencer R.M.C., Zelaznik H.N., Diedrichsen J. (2002). The cerebellum and event timing. Ann. N. Y. Acad. Sci..

[46] Penhune V.B., Zatorre R.J., Evans A.C. (1998). Cerebellar contributions to motor timing: A PET study of auditory and visual rhythm reproduction. J. Cogn. Neurosci..

[47] Sakai K., Hikosaka O., Miyauchi S., Takino R., Tamada T., Iwata N.K., Nielsen M. (1999). Neural representation of a rhythm depends on its interval ratio. J. Neurosci..

[48] Schubotz R.I., Friederici A.D., Von Cramon D.Y. (2000). Time perception and motor timing: A common cortical and subcortical basis revealed by fMRI. Neuroimage.

[49] Thaut M.H., Stephan K.M., Wunderlich G., Schicks W., Tellmann L., Herzog H., McIntosh G.C., Seitz R.J., Hömberg V. (2009). Distinct cortico-cerebellar activations in rhythmic auditory motor synchronization. Cortex.

[50] Chen J.L., Zatorre R.J., Penhune V.B. (2006). Interactions between auditory and dorsal premotor cortex during synchronization to musical rhythms. Neuroimage.

[51] Chen J.L., Penhune V.B., Zatorre R.J. (2008). Listening to musical rhythms recruits motor regions of the brain. Cereb. Cortex.

[52] Bengtsson S.L., Ullén F., Ehrsson H.H., Hashimoto T., Kito T., Naito E., Forssberg H., Sadato N. (2009). Listening to rhythms activates motor and premotor cortices. Cortex.

[53] Koelsch S., Siebel W.A. (2005). Towards a neural basis of music perception. Trends Cogn. Sci..

[54] Maess B., Koelsch S., Gunter T.C., Friederici A.D. (2001). Musical syntax is processed in Broca’s area: An MEG study. Nat. Neurosci..

[55] Uddén J., Bahlmann J. (2012). A rostro-caudal gradient of structured sequence processing in the left inferior frontal gyrus. Philos. Trans. R. Soc. B Biol. Sci..

[56] Friederici A.D. (2001). Syntactic, prosodic, and semantic processes in the brain: Evidence from event-related neuroimaging. J. Psycholinguist. Res..

[57] Coull J.T., Vidal F., Nazarian B., Macar F. (2004). Functional anatomy of the attentional modulation of time estimation. Science.

[58] Harrington D.L., Zimbelman J.L., Hinton S.C., Rao S.M. (2009). Neural modulation of temporal encoding, maintenance, and decision processes. Cereb. Cortex.

[59] Coull J.T., Davranche K., Nazarian B., Vidal F. (2013). Functional anatomy of timing differs for production versus prediction of time intervals. Neuropsychologia.

[60] Battelli L., Cavanagh P., Martini P., Barton J.J.S. (2003). Bilateral deficits of transient visual attention in right parietal patients. Brain.

[61] Battelli L., Walsh V., Pascual-Leone A., Cavanagh P. (2008). The ‘when’ parietal pathway explored by lesion studies. Curr. Opin. Neurobiol..

[62] Oliveri M., Koch G., Salerno S., Torriero S., Gerfo E.L., Caltagirone C. (2009). Representation of time intervals in the right posterior parietal cortex: Implications for a mental time line. Neuroimage.

[63] Grahn J.A., Rowe J.B. (2012). Finding and feeling the musical beat: Striatal dissociations between detection and prediction of regularity. Cereb. Cortex.

[64] Sihvonen A.J., Särkämö T., Rodríguez-Fornells A., Ripollés P., Münte T.F., Soinila S. (2019). Neural architectures of music—Insights from acquired amusia. Neurosci. Biobehav. Rev..

[65] Ivry R.B., Schlerf J.E. (2008). Dedicated and intrinsic models of time perception. Trends Cogn. Sci..

[66] Mauk M.D., Buonomano D.V. (2004). The neural basis of temporal processing. Annu. Rev. Neurosci..

[67] Buhusi C.V., Meck W.H. (2005). What makes us tick? Functional and neural mechanisms of interval timing. Nat. Rev. Neurosci..

[68] Gibbon J., Church R.M., Meck W.H. (1984). Scalar timing in memory. Ann. N. Y. Acad. Sci..

[69] Matell M.S., Meck W.H. (2004). Cortico-striatal circuits and interval timing: Coincidence detection of oscillatory processes. Cogn. Brain Res..

[70] Karmarkar U.R., Buonomano D.V. (2007). Timing in the absence of clocks: Encoding time in neural network states. Neuron.

[71] Merchant H., Harrington D.L., Meck W.H. (2013). Neural basis of the perception and estimation of time. Annu. Rev. Neurosci..

[72] Merchant H., Zarco W., Prado L. (2008). Do we have a common mechanism for measuring time in the hundreds of millisecond range? Evidence from multiple-interval timing tasks. J. Neurophysiol..

[73] Konoike N., Mikami A., Miyachi S. (2012). The influence of tempo upon the rhythmic motor control in macaque monkeys. Neurosci. Res..

[74] Hattori Y., Tomonaga M., Matsuzawa T. (2013). Spontaneous synchronized tapping to an auditory rhythm in a chimpanzee. Sci. Rep..

[75] Patel A.D., Iversen J.R., Bregman M.R., Schulz I. (2009). Experimental evidence for synchronization to a musical beat in a nonhuman animal. Curr. Biol..

[76] Rouse A.A., Cook P.F., Large E.W., Reichmuth C. (2016). Beat Keeping in a Sea Lion As Coupled Oscillation: Implications for Comparative Understanding of Human Rhythm. Front. Neurosci..

[77] Zarco W., Merchant-Nancy H., Prado L., Méndez J.C. (2009). Subsecond timing in primates: Comparison of interval production between human subjects and rhesus monkeys. J. Neurophysiol..

[78] Merchant-Nancy H., Pérez O., Bartolo R., Méndez J.C., Mendoza G., Gámez J., Yc K., Prado L. (2015). Sensorimotor neural dynamics during isochronous tapping in the medial premotor cortex of the macaque. Eur. J. Neurosci..

[79] Merchant H., Pérez O., Zarco W., Gámez J. (2013). Interval tuning in the primate medial premotor cortex as a general timing mechanism. J. Neurosci..

[80] Merchant H., Zarco W., Pérez O., Prado L., Bartolo R. (2011). Measuring time with different neural chronometers during a synchronization-continuation task. Proc. Natl. Acad. Sci. USA.

[81] Taut M. (2006). Rhythm, Music, and the Brain: Scientific Foundations and Clinical Applications (Studies on New Music Research).

[82] Nombela C., Hughes L.E., Owen A.M., Grahn J.A. (2013). Into the groove: Can rhythm influence Parkinson’s disease?. Neurosci. Biobehav. Rev..

